# Asynchronous coding in neuronal networks

**DOI:** 10.1186/1471-2202-15-S1-P26

**Published:** 2014-07-21

**Authors:** Eric S Kuebler, Jean-Philippe Thivierge

**Affiliations:** 1 School of Psychology, University of Ottawa, Ottawa, Ontario, Canada, K1N 6R5

## 

**Figure 1:**
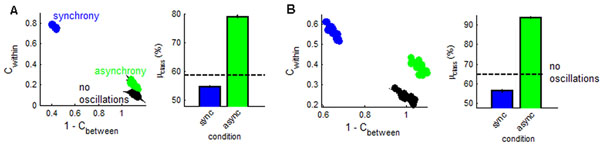
**Reliability of responses and percentage of accurately classified stimuli. A. ***Left*: Scatter plot of reliability and discriminability in network responses to stimuli. The x-axis shows discriminability, measured as 1-**C***_between_*. The y-axis shows reliability **C***_within_*. Green circles: asynchronous network (frequency 10 Hz & amplitude 2 µA). Blue circles: synchronous network (10 Hz – 2 µA). Black circles: network without oscillations. *Right***:** Percentage of accurately classified stimuli. Dashed black line represents networks without oscillations. **B**. Same as A, but strictly for cells that directly received a stimulus.

Recordings of neuronal networks in cortex show evidence of asynchronous – or out-of-phase – activity. Cells are known to generate asynchronous outputs despite strongly shared synaptic inputs [1], yet the computational benefits of this coding remain unclear. By comparison, synchronized activity in neuronal networks has been reported in a broad range of experiments [2], with a proposed functional role of enhancing the reliability of responses to stimuli [3]. Starting from a model of randomly connected leaky integrate-and-fire neurons (*N* = 1000), we injected sub-threshold oscillations that were either asynchronous or synchronous, resulting in time-lagged or zero-lag correlations in spiking activity, respectively. We then examined the ability of neurons to respond reliably and discriminately to stimuli (large depolarizing events) delivered to random subsets of the population. We measured reliability (**C***_within_*) by examining the correlations between responses to the same stimulus, and discriminability (**C***_between_*) as the correlation between responses to different stimuli. Asynchronous networks responded with moderate reliability and high discriminability (green circles – Figure 1). By comparison, synchronous networks yielded the opposite effect and led to high reliability and low discriminability (blue circles – Figure 1). To provide a proof of principle that asynchronous networks could accurately classify stimuli, we designed a simple classification criterion based on the reliability of responses to stimuli. Results of this analysis show that asynchronous coding was more useful in classifying stimuli than synchronous networks (Figure 1A-B). We found that asynchronous coding may be especially beneficial to the subset of cells that was directly stimulated by an input (Figure 1B). The trade-off observed between reliability and discriminability may be continuous in nature – neurons receiving a hybrid combination of synchronous with asynchronous activity (in equal parts) were more reliable than asynchronous neurons and better at discriminating between stimuli than synchronous neurons*.* In sum, our work highlights a novel form of trade-off between asynchrony and synchrony. We suggest that living neuronal networks may take advantage of both forms of coding depending on the context and requisites of information processing.
